# Metagenomic Analysis of Virioplankton of the Subtropical Jiulong River Estuary, China

**DOI:** 10.3390/v8020035

**Published:** 2016-02-02

**Authors:** Lanlan Cai, Rui Zhang, Ying He, Xiaoyuan Feng, Nianzhi Jiao

**Affiliations:** 1 State Key Laboratory of Marine Environmental Science, Institute of Marine Microbes and Ecospheres, Xiamen University (Xiang’an), Xiamen 361005, China; cailanlan@stu.xmu.edu.cn; 2 State Key Laboratory of Microbial Metabolism, School of Life Sciences and Biotechnology, Shanghai Jiao Tong University, Shanghai 200240, China; heying1982@sjtu.edu.cn (Y.H.); asdfeng131@sjtu.edu.cn (X.F.)

**Keywords:** virus, estuary, virome, next-generation sequencing

## Abstract

Viruses are the most abundant biological entities in the oceans, and encompass a significant reservoir of genetic diversity. However, little is known about their biodiversity in estuary environments, which represent a highly dynamic and potentially more diverse habitat. Here, we report a metagenomic analysis of the dsDNA viral community from the Jiulong River Estuary (JRE), China, and provide a comparative analysis with other closely related environments. The results showed that the majority of JRE virome did not show any significant similarity to the database. For the major viral group (*Caudovirales*) detected in the sample, *Podoviridae* (44.88%) were the most abundant family, followed by *Siphoviridae* (32.98%) and *Myoviridae* (17.32%). The two most abundant viruses identified in the virome were phages HTVC010P and HMO-2011, which infect bacteria belonging to marine SAR11 and SAR116 clades, respectively. Two contigs larger than 20 kb, which show similar overall genome architectures to *Celeribacter* phage P12053L and *Thalosomonas phage BA3*, respectively, were generated during assembly. Comparative analysis showed that the JRE virome was more similar to marine viromes than to freshwater viromes, and shared a relative coarse-grain genetic overlap (averaging 14.14% ± 1.68%) with other coastal viromes. Our study indicated that the diversity and community structure of the virioplankton found in JRE were mainly affected by marine waters, with less influence from freshwater discharge.

## 1. Introduction

Over the last three decades, viruses, particularly those infecting bacteria (bacteriophages), have received increasing recognition for their importance in marine environments. This recognition is based upon four main lines of evidence. First, viruses are the most abundant organisms in the oceans, comprising approximately 94% of the nucleic acid-containing particles, and encompassing a significant reservoir of genetic diversity [1,2]. Second, viruses keep microbial numbers at values less than the carrying capacity of the system through viral lysis. This is well known as the “kill the winner” hypothesis, and plays an important role in shaping microbial population structures and mediating genetic exchange between hosts [3,4]. Third, viruses are able to reprogram the metabolic output of their hosts through the expression of virally encoded auxiliary metabolic genes, which play critical roles in facilitating biochemical and metabolic processes [5,6]. For example, cyanophages can transcribe and express photosynthesis genes during lytic infection, helping to maintain photosynthesis during the phage lytic cycle and thereby promote viral propagation [7,8]. Finally, referred to as the “viral shunt”, the lytic processes of the host microbial cells infected by marine viruses will release dissolved organic matters into the water, which can be used by other heterotrophic bacteria. This means that virus-mediated mortality increases net respiration, dissolved organic matter recycling and nutrient transformation in aquatic systems [2,9,10,11].

Viral metagenome (virome) analysis overcomes the limitation of traditional methods and circumvents the drawback that there are no universal signature genes that can be used as phylogenetic markers to assess viral diversity. It has emerged as a promising method not only for studying viral diversity in a wide range of research samples, such as marine environments [12,13], fresh waters [14,15,16,17], plants [18], feces [19], blood [20], animal tissue [21], and other specialized habitats [22,23] but also for discovering novel viruses [13,24]. Combined findings from previous virome surveys suggest that viruses carry a tremendous genetic diversity. The majority (usually, 60%–99%) of sequences in viromes from any environment do not share homology with databases [25,26,27]. Recently, the *Tara* Oceans Expedition (2009–2013) carried out a global survey of viromes by collecting a wide variety of samples from the surface, deep chlorophyll maximum and mesopelagic zones of seven oceans and seas. The large-scale dataset confirms a high local viral diversity, which may result from localized pockets of viruses under small-scale environmental constraints [28]. However, so far, the majority of virome analyses have focused on marine or fresh waters and, to a lesser extent, transition zones, such as estuaries. Viral communities in estuary environments are likely to be more diverse than those of their marine and freshwater counterparts. The mixing zone between marine tidal and river outflow, together with nutritional surplus, is a highly dynamic environment that can trigger physiological, genetic, and ecological changes in microbes, and thus exert a potentially profound effect on food web interactions and estuarine biogeochemical cycling [29,30]. The Pacific Ocean Viromes dataset has revealed decreased functional richness with distance from shore in surface waters [31]. A previous study of viral diversity in a gradient of anthropogenic impacts demonstrated that the mixed sample produced the highest viral genotype richness [14]. Meanwhile, estuaries are of great ecological significance as they often serve as spawning sites for fish and shellfish species, and are the connection between watersheds and oceans. In spite of their significant importance, relatively little is known about the composition and structure of viral communities in estuaries compared with other aquatic environments.

The Jiulong River is the second longest river in Fujian Province, southeast China. The Jiulong River estuary (JRE) is a typical subtropical macro-tide estuary connected with Xiamen Sea harbor and on the southwest coast of the Taiwan Strait. A recently published study that investigated the distribution and diversity of bacterioplankton communities of Xiamen Sea harbor found that the sampling site near the JRE exhibited the highest bacterial diversity, and was significantly influenced by total nitrogen and total phosphorus [32]. Because of the nature of the host-specific interactions, we could expect the composition of viruses (most of which are phages) to follow that of the bacterial community. In this study, we present a double-stranded DNA (dsDNA) viral metagenome dataset from the JRE. Given the high level of plasticity of viral genomes, the current virome analysis improves our knowledge of aquatic viral ecology and assists in identifying viral populations specific to subtropical estuarine environment.

## 2. Materials and Methods

### 2.1. Sample Collection and Sequencing

About 150 L of surface water were collected from the JRE, China (118°16′54.56″ E, 24°30′17.00″ N). No specific permits or permissions were required for the sampling. Temperature, salinity, and pH were measured *in situ* with a YSI Professional Plus multiparameter meter (YSI Incorporated, Yellow Springs, OH, USA). Samples were processed immediately according to Thurber *et al.* [33]. Briefly, the samples were filtered through a 3-μm filter to remove large particles. Two-step tangential flow filtration (TFF) was then used to concentrate viruses. First, a 0.22-μm pore size filter (0.5 m^2^ Pellicon cartridge, Millipore Corp., Billerica, MA, USA) was used to remove bacteria and small eukaryotes. Viral particles were further concentrated using a TFF 30-kDa cartridge to a final volume of 2 L. The samples were precipitated using polyethylene glycol (PEG-8000) (10% w/v) and incubated at 4 °C overnight. Virus-like particles from the PEG pellet were purified using cesium chloride gradient ultracentrifugation (1.7, 1.5, and 1.35 g/mL layers). Virus-like particles were collected from the 1.35–1.5 g/mL fraction and confirmed by epifluorescence microscopy. After being treated with DNase I and RNase A at 4 °C for 1 h, the purified virus-like particles were treated with Proteinase K, EDTA (0.5 M) and 10% SDS at 55 °C for 3 h. DNA was extracted using phenol/chloroform/isoamylol method. To test for contaminating bacterial DNA, viral nucleic acids were screened by 16S rDNA PCR with 27F/1492R universal primers. After DNA quality and quantity checks by DNA agarose gel electrophoresis, DNA was sequenced using Roche 454 GS FLX system (Roche 454 Life Sciences, Branford, CT, USA).

### 2.2. Microbial Enumeration

For microbial counts, 2-mL aliquots were fixed in a final concentration of 0.5% glutaraldehyde at 4 °C for 20 min, then stored at −80 °C after snap freezing in liquid nitrogen. Viral and heterotrophic bacterial abundances were determined by flow cytometry (FCM) according to Marie *et al.* [34] and Bruaassrd [35]. Briefly, the fixed frozen samples were thawed at room temperature and stained with SYBR Green I (Invitrogen, Carlsbad, CA, USA). The stained particles were enumerated using a flow cytometer (Epics Altra II, Beckman Coulter, Miami, FL, USA) at event rates of 50–200 particles/s (bacteria) or 100–300 particles/s (virus). The autotrophic picoplankton abundance was determined according to the method of Jiao *et al.* [36]. Picoeukaryotes and *Synechococcus* were distinguished according to their positions in the cytometric plots. The analyses of microbial abundance by FCM for each sample were repeated twice. The data were analyzed with EXPO^TM^32 MultiCOMP software [37].

### 2.3. Bioinformatics Analysis

A series of filtering and trimming steps were undertaken to remove low quality reads using cd-hit 454 [38] and Mothur v.1.33.0 [39]. Clean reads were subsequently assembled using GS De novo Assembler (Newbler v2.5, Roche 454 Life Sciences) with default parameters. Both clean reads and assembled contigs from the JRE virome were uploaded to Metavir [40], an online tool for analyzing viral genomic data, and the Meta Genome Rapid Annotation using Subsystem Technology (MG-RAST) server [41], a general metagenomic webserver, with MG-RAST accession number 4,527,838.3. Metavir performs taxonomic composition analysis based on BLASTx searches against the National Center for Biotechnology Information (NCBI) viral refseq database. MG-RAST generates taxonomic assignments based on BLASTx searches against the M5NR database and functional categories based on BLASTx searches against the SEED-Subsystem database. Contigs larger than 20 kb obtained from the above assembly were examined further. Putative open reading frames (ORFs) within each contig were predicted using GeneMarkS [42], and then searched against the NCBI non-redundant protein database using BLASTp. BLAST homologies having an *E*-value less than 10^−3^ were considered putative hits. Comparisons to publicly available aquatic viromes were carried out in Metavir by performing tBLASTx comparisons after data normalization. To obtain an in-depth comparison of the virome obtained in this study with closely related datasets, we downloaded eight Pacific Ocean coastal surface virome datasets (PCSVs) from a previous study, including samples GFS, GDS, LJ4S, MACS, SFSS, SFDS, SFCS, and STCS [31]. All datasets were subsampled to a common depth (the minimum number of sequences in the GFS virome; 82,739), and then trimmed to 100-bp to homogenize the sequence length and effectively normalize the sampling effort. A cross-tblastx (*E*-value < 10^−3^) was undertaken to study the degree of putative coarse-grain genetic overlap between these environments [17].

## 3. Results

### 3.1. Environmental Characteristics

**Figure 1:**
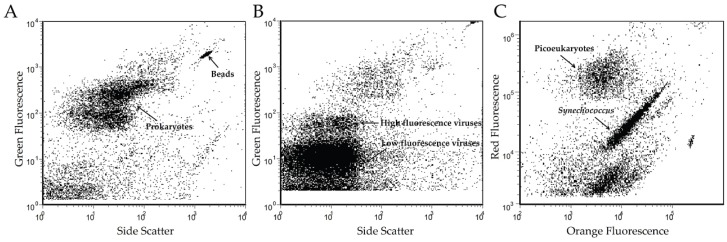
Cytometric plot graph of bacterioplankton (**A**), virioplankton (**B**) and picophyplankton (**C**) populations of sample from the JRE.

As an estuary environment, the JRE undergoes mixing of the Jiulong River outflow with tidal water from the Xiamen Sea harbor. The salinity of the JRE was 25.50, with a pH value of 7.88 and a sea surface temperature of approximately 23.3 °C. The abundance of virioplankton in the JRE, as estimated by FCM, was 3.38 × 10^7^ particles/mL, which was more than an order of magnitude higher than that of bacterioplankton (2.21 × 10^6^ particles/mL). The abundances of picoeukaryotes and *Synechococcus* were estimated as 2.09 × 10^4^ cells/mL and 7.03 × 10^4^ cells/mL, respectively (Figure 1).

### 3.2. Overview of the JRE Virome

**Figure 2:**
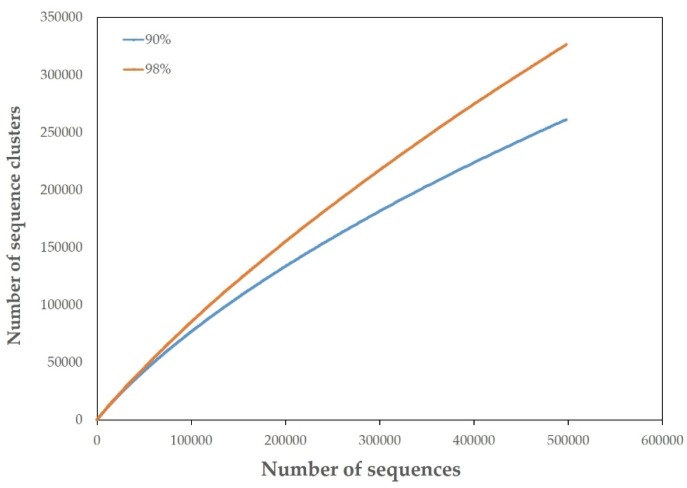
Rarefaction curve of the JRE virome generated by Metavir. Clustering were set at 90% and 98% similarities.

Pyrosequencing of the JRE virome resulted in 527,507 raw reads, with an average length of 495 bp. A total of 498,957 reads with a 505-bp average length were generated after filtering and trimming. The average G + C content was 42.6% for clean data. Based on 90% sequence identity, the rarefaction curve generated by Metavir showed approximately 260,000 sequencing clusters. The number of clusters increased to 325,000 at 98% similarity (Figure 2). According to the current phage taxonomy, viral isolates are grouped into the same species at nucleotide identity levels of 90%–95% [43]. Given an average phage genome length of 50,000 bp and an average read length of 500 bp [12,44], the viral richness of JRE metavirome was estimated to be between 2600 and 3250 (# clusters/(genome length/read length)), which is almost twice higher than that of a recently published hypolithic virome [43].

### 3.3. Taxonomic Composition

**Figure 3:**
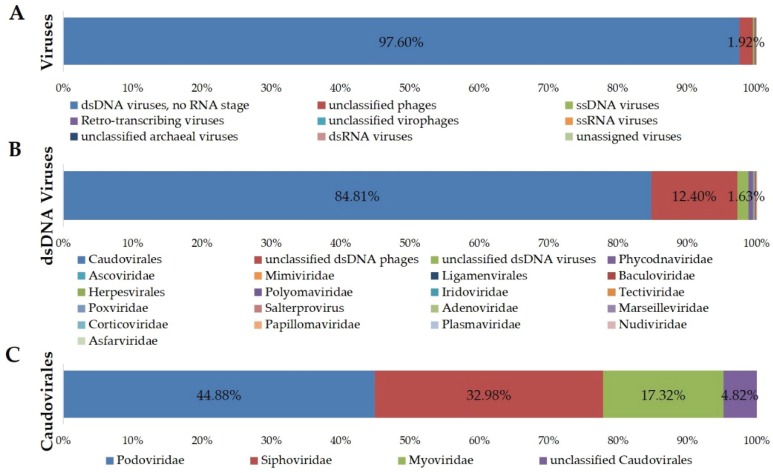
Taxonomic composition of the JRE virome, based on BLAST comparison with the NCBI Refseq complete viral genomes protein sequences database (release of 5 January 2015) by Metavir with an *E*-value threshold of 10^−5^.

A total of 498,957 reads with a 505-bp average length were used for analysis of the JRE viral taxonomic composition. According to the MG-RAST annotation, 40.7% of reads had homology to known sequences. Reads were also assessed by Metavir for in-depth analysis of viral taxonomic composition using the GAAS tool, which normalizes the composition plot according to genome lengths [45], with an *E*-value threshold of 10^−5^. As with most viromes, the vast majority of reads from the JRE virome did not show any significant sequence similarity to the database. Only 21.42% of the reads produced significant hits. Most (97.60%) of the sequences with significant hits were recognized as belonging to dsDNA viruses (Figure 3), while similarities to single-stranded DNA phages and RNA viruses were also observed. The majority (84.81%) of sequences showing similarity to dsDNA viruses belonged to the order *Caudovirales*, with *Podoviridae* being the most abundant family (44.88%), followed by *Siphoviridae* (32.98%) and *Myoviridae* (17.32%). Furthermore, 4.82% of the sequences shared identity with “unclassified *Caudovirales*” sequences. Other dsDNA viruses identified at an abundance of 0.05%–1% included *Phycodnaviridae*, *Ascoviridae* and *Mimiviridae*. The rank-abundance curve for the JRE virome was characterized by a small number of abundant genomes and a large number of genomes with a relative abundance <1%. Among the 13 genomes with a relative abundance >1%, the five most abundant were identified as *Pelagibacter* phage HTVC010P (5.08%), *Puniceispirillum* phage HMO-2011 (3.91%) and *Thalassomonas* phage BA3 (2.07%), three *Podoviridae* viruses, and two members of unclassified dsDNA phages, *Pseudomonas* phage PA11 (2.64%) and *Celeribacter* phage P12053L (2.12%). Phages infecting enterobacteria, S*taphylococcus* species, and other pathogens infecting mammalian hosts were detected at >0.5% abundance. Two eukaryotic viruses, *Tetraselmis viridis* virus SI1 and *Dunaliella viridis* virus SI2 were also identified at relative abundances of 0.59% and 0.50%, respectively.

### 3.4. Functional Analysis

**Figure 4:**
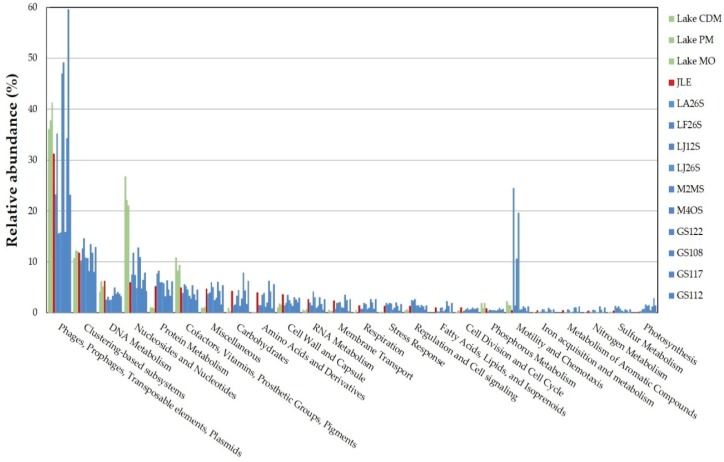
Comparison of metabolic subsystems of the JRE virome with other aquatic viromes. The coding sequences were compared with SEED database using subsystems in MG-RAST. Metabolic subsystems from the JRE virome are colored by red. Green represents functional categories from temperate lakes (stations CMD, PM, and MO) [14]. Blue represents functional categories from the Pacific Ocean (stations LA26S, LF26S, LJ12S, LJ26S, M2MS, and M4OS) [31], and the Indian Ocean (stations GS122, GS108, GS117, and GS112) [46].

Metabolic subsystems in the JRE virome were annotated using MG-RAST. The data were compared to subsystems using a maximum *E*-value of 1e^−5^ and a minimum identity of 60%. The database search resulted in 49,387 functional hits, the majority (31.26%) of which belonged to the subsystem “Phages, prophages, transposable elements, plasmids” with phage structural, integration/excision and DNA metabolism-related proteins. Other highly represented (>5%) functional categories included “Clustering-based subsystem”, “DNA metabolism”, “Nucleotides and nucleosides” and “Protein metabolism” (Figure 4).

The functional categories of the JRE virome were then compared with those of other aquatic viromes, including temperate lakes (stations CMD, PM, and MO) [14], the Pacific Ocean (stations LA26S, LF26S, LJ12S, LJ26S, M2MS, and M4OS) [31], and the Indian Ocean (stations GS122, GS108, GS117, GS112) [46] on the MG-RAST server. Based on the comparison, we determined that subsystems related to “Nucleotides and nucleosides” and “Cofactors, vitamins, prosthetic groups, and pigments” were significantly over-represented in lake viromes compared with the JRE virome and other marine counterparts (Figure 4). However, some functions, including “Protein metabolism”, “Miscellaneous”, and “Carbohydrate” were more highly represented in the JRE virome and viromes from other marine environments.

### 3.5. Contig Analysis

Assembly using Newbler yielded 13,278 contigs, with an average length of 1126 bp accounting for a total of 15,729,985 bp for JRE virome. Because of the input restriction that only sequences longer than 300 bp are considered for projects of type contigs by Metavir, 10,246 contigs longer than 300 bp were selected for analysis. In assessing the composition of the assembled contigs, 39.15% of the contigs showed similarity to known sequences. A total of 31,476 genes were predicted, with a high proportion (78.4%) of predicted ORFs showing no significant homology to sequences present in the databases.

**Figure 5:**
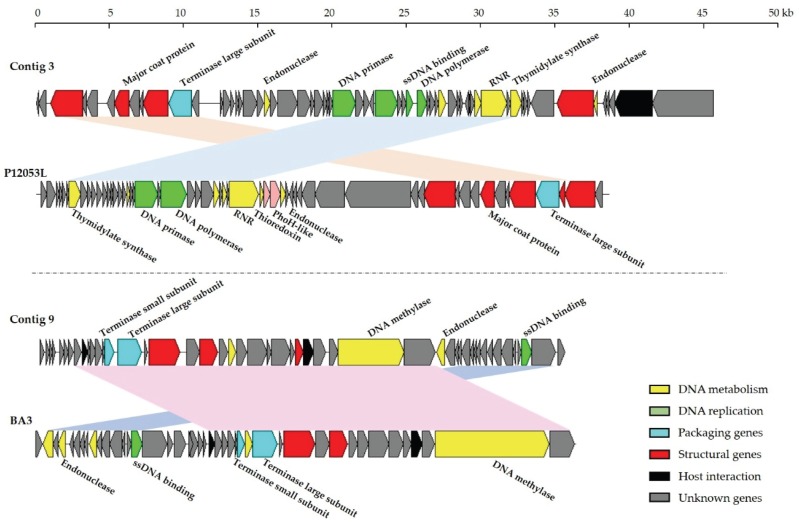
Comparisons of contigs 3 and 9 with *Celeribacter* phage P12053L and *Thalosomonas phage BA3*, respectively. ORFs are depicted by leftward or rightward oriented arrows according to the direction of transcription. Colored arrows denote putative functions assigned by Blastp similarity. Homologous ORFs are connected by shadowing.

Of the 8367 contigs >500 bp, 28 contigs >20 kb were assembled, with the largest being 62 kb. Only two contigs produced significant hits (*E*-value < 10^−3^) to complete viral genomes in the publicly available database. The two contigs were 46,070 bp (contig 3) and 36,247 bp (contig 9) long, with the overall G + C content 44.70% and 40.35%, respectively. Sixty-two ORFs were predicted by GeneMarkS within contig 3, of which 15 showed similarity to *Celeribacter* phage P12053L (Figure 5), a marine member of the unclassified *Caudovirales* group [47], indicating that this contig almost certainly belonged to the order *Caudovirales*. The majority of genes in contig 3 for which a function could be assigned (11 of 17) encoded proteins related to DNA metabolism and replication, including endonuclease, DNA primase, DNA polymerase, single-strand DNA binding protein, thymidylate synthase and ribonucleotide reductase. Four ORFs were predicted to encode proteins involved in the structure and assembly of virions. The translated product of an ORF identified as the terminase large subunit gene showed 80% amino acid similarity to the corresponding protein of *Celeribacter* phage P12053L. Lysozyme genes were also predicted within contig 3. The genome maps showed that contig 9 present similar overall genome organization to that of *phage BA3* (Figure 5), which lyses the coral pathogen *Thalassomonas loyana*. In total, 61.2% of ORFs identified (30 of 49) from contig 9 showed similarity to those of *phage BA3*, with the corresponding proteins showing 28%–100% amino acid identity.

### 3.6. Comparison with Other Viromes

**Figure 6:**
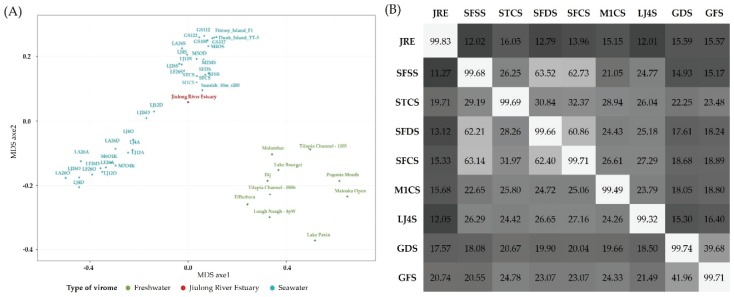
(**A**) MDS plots of freshwater (green) and marine (cyan) publicly available viromes with the JRE virome (red) using Metavir; (**B**) Coarse-grain genetic overlap between the JRE virome and PCSVs. The heat-plot represents the results of cross-tblastx comparisons between samples. Values (% of total reads with hits) with *E*-value ≤ 10^−3^ are labeled within the boxes. Horizontal labels represent viromes acting as query, and vertical labels represent viromes acting as reference.

BLAST comparisons of the JRE virome with previously published aquatic viromes were carried out to examine the similarities between different aquatic viral communities. The results revealed that aquatic viromes could be classified into two representative groups, freshwater viromes and marine viromes (Figure 6A). Generally, the JRE virome clustered with the marine viromes, and was closely related to the Pacific Ocean surface samples from previous study [31]. To obtain a deeper comparison of related datasets, the JRE virome was further compared with eight PCSVs. Comparison of the dominant members of the JRE virome with the PCSVs showed that most viral genotypes were shared, but that there were large differences in the relative abundance of particular species between the samples. In addition, two *Pseudomonas* phages infecting *P. aerugin*osa, an opportunistic human pathogen, were exclusively found in the JRE sample. The results of the cross-tblastx analysis indicated that the JRE virome showed a coarse-grain genetic overlap of 14.14% ± 1.68% with the PCSVs, with the highest relative overlap observed with the virome of sample STCS, followed by GDS and GFS (Figure 6B). Samples SFSS, STCS, SFDS, and SFCS were derived from the same coastal surface water from Scripps Pier, San Diego, CA, USA [31]. Sites GDS and GFS, corresponding to the Great Barrier Reef, Australia, were under the influence of the Tully River, which appeared to explain the relatively high genetic overlap with the JRE virome.

## 4. Discussion

### 4.1. The JRE Virome

Consistent with most other viromes examined [12,14,43,46,48], the investigation of dsDNA viral diversity in the JRE showed that the majority of sequence reads were classified as unknown, with only 21.42% having known virus matches. This is in line with identification rates for the value of the other aquatic dsDNA metaviromes [14,31,46,49], which range from 6.75% to 28.67%. These findings support the hypothesis that much of the global viral diversity remains uncharacterized. Analysis of taxonomic composition for JRE virome showed most viruses belonged to the order *Caudovirales*, with *Podoviridae* representing the major fraction, followed by *Siphoviridae* and *Myoviridae*. In addition, since the rarefaction curve of the JRE virome did not tend to approach a horizontal asymptote, the viral richness of the JRE estuary was potentially higher than that revealed here.

Based on clustering analysis in which viromes were compared to each other via Metavir through BLASTx, viral communities appeared to group according to habitat, with viromes from freshwater and marine samples forming distinct clusters. Similar results were also obtained from previous studies [14,16]. This reflected unique virome signatures contained in contrasting environments (seawater *vs.* freshwater). Therefore, viral communities in estuarine environments are expected to be more diverse due to the mixing of seawater and freshwater, the resuspension of sediments and the interference of human activities. Previous virome investigation of several environmental transects already suggested that surface viral richness decreased with coast-open ocean gradients [31], which mirrored the distribution pattern of their hosts (mainly bacteria).

### 4.2. Impacts of Marine Water on the Composition of the JRE Virome

Clustering analysis showed that the JRE virome clustered with other marine viromes and was closely related to the PCSVs, suggesting that it was mainly affected by marine waters. The JRE virome also shared a significant genetic overlap with viromes from costal and estuarine areas. Despite an overall similarity in the viral genotypes of the viromes examined, there were large differences in the relative abundance of particular families between the JRE virome and the PCSVs. This finding is consistent with the results of a previous study, which indicated that the change in abundance of the most common members is what sets different assemblages apart, rather than the exclusion of different viral genomes [13]. This idea forms the basis of a theoretical prediction made for marine virus communities, termed the “Seed-Bank model” [50].

Obvious marine signatures were observed following deeper inspection of phage genotypes. Viruses known to infect typical marine bacteria, such as *Proteobacteria* and *Bacteroidetes*, were widely present in the JRE sample. For example, the most abundant virus identified in the JRE virome was *Pelagibacter* phage (*Pelagiphage*) HTVC010P, which infect “*Candidatus* Pelagibacter ubique” of the SAR11 clade, the most abundant bacterium in surface seawater around the world [51]. In a more extensive metagenomic investigation including samples from coastal and open ocean areas, *Pelagiphage* HTVC010P was proposed as one of the most abundant virus subfamilies in the biosphere, in which 38.8% of successfully assigned reads were assigned to HTVC010P [52]. The second-most abundant virus genome was phage HMO-2011, a phage infecting “*Candidatus* Puniceispirillum marinum” of the SAR116 clade. Phage HMO-2011 was firstly isolated from the East Sea and was revealed to be widely distributed in the ocean, accounting for 10.3%–25.3% of reads in seven viromes from the coastal and open area of the Pacific and Indian Oceans [53]. Our study demonstrates that *Pelagiphage* HTVC010P and phage HMO-2011 may be abundant in estuarine environments as well.

In general, the prevalence of specific viral populations follows that of their hosts. The composition of the metavirome sequenced in the current study is consistent with the results of 16S rRNA gene analysis performed on samples from the same habitat [32]. The most dominant bacterial phylum was *Proteobacteria*, followed by *Bacteroidetes*, which is consistent with our assignment of the virome. At the operational taxonomic unit level, the most abundant bacterial sequences were affiliated with the SAR11 clade. Therefore, the prevalence of *Pelagiphage* HTVC010P and phage HMO-2011 sequences in the JRE virome corroborates the high abundance of their marine bacterial hosts.

Further evidence of the impact of marine water on the composition of the JRE virome was obtained from contig analysis. Two of the assembled contigs showed significant similarity to the complete genomes of *Celeribacter* phage P12053L and *Thalosomonas* phage BA3, respectively. *Celeribacter* phage P12053L, a lytic bacteriophage infecting bacteria of the the *Roseobacter* clade, was isolated from seawater collected off the coast of the Yellow Sea [47]. The Yellow Sea is close to the environment sampled in this study, confirming *Celeribacter* phage P12053L as a significant contributor to viral communities in coastal surface waters, and the high abundance of *Roseobacteria* in the ocean. *Thalosomonas* phage BA3, which lyses the coral pathogen *Thalassomonas loyana*, has previously been isolated from the Red Sea [54]. The presence of these two contigs in the JRE virome suggests the prevalence of *Celeribacter* phage P12053L and *Thalosomonas* phage BA3 in the JRE, and supports the importance of marine microbial populations in shaping the estuarine virome.

### 4.3. Impacts of River Outflow on the Composition of the JRE Virome

In general, relatively few impacts of river outflow were found in JRE virome, which agreed with the general environmental characteristics of our sampling station. The salinity and other environmental factors indicated that the sampling site was more similar to typical marine environments. Therefore, it is not surprising that only a few functional categories were consistent with lake viromes, showing higher relative abundance than in marine viromes. However, two *P. aeruginosa* phages were exclusively found in the JRE sample when compared with the PCSVs. *P. aeruginosa* is an opportunistic pathogen that is important in the etiology of many infectious diseases of humans, and is widely distributed in non-marine environments (e.g., soil, ground water, sewage, the mammalian gut). The apparent distribution of *P. aeruginosa* in the marine system has been restricted to river outfalls and shorelines [55,56,57]. Therefore, we deduced that *Pseudomonas* phages in the JRE virome probably originated from Jiulong River discharge. Phages infecting enterobacteria and other mammalian pathogens were also found in the JRE virome. Similar to their pathogen hosts, the occurrence of these non-marine phages in an estuary virome likely results from freshwater transfer from the river, which is a site of intense human activities.

The duality of the JRE virome can be explained by idea that the JRE is a sort of mixing chamber that receives Jiulong River outflow with the dash of marine water. Similar patterns for their hosts were frequently observed in estuarine area [32,58,59]. Previous research showed that the dynamics of viral abundance in the Yangtze River estuarine area were characterized by distinct seasonal and spatial variations caused by natural forces and anthropogenic impacts [58]. In addition, two eukaryotic viruses, SI1 and SI2, were identified in the current study. These viruses infect the eukaryotic microalgae *Tetraselmis viridis* Rouch and *Dunaliella viridi*. These two microalgae are commonly found in a wide range of habitats with varying degrees of salinity: from fresh water to salt lakes containing sodium chloride at concentrations up to saturation. The presence of these viruses in the JRE virome might reflect the adaptation of the eukaryotic microbial population to dynamic estuary environments with large temporal and spatial fluctuations of environmental parameters such as salinity.

### 4.4. Methodological Considerations and Limitations

Metagenomic analysis has become an invaluable tool to explore total viral taxonomic composition and diversity from environmental samples. However, the virome procedure from sample collection, sequencing preparation to bioinformatics analysis is experimental and informatics-challenging, which may induce biases in estimating viral diversity.

Due to the small individual size and biomass, virus enrichment steps are necessary to obtain sufficient experimental materials [60]. As with the majority of viromes published, a 0.22-μm TFF step was used in this study to remove cells. Large viruses such as *Phycodnaviridae* were likely removed during this process, resulting in the low representation in the virome. Recently, a new chemistry-based concentration method, FeCl_3_ precipitation, has emerged and showed nearly complete viral recovery, which may be widely applied for viral concentration in the future [61].

Although the prevalence of *Caudovirales* sequences was observed in most aquatic viromes, previous research on global viral morphology through the *Tara* Oceans Expedition has revealed that non-tailed viruses numerically dominated the upper oceans [62]. Non-tailed viruses are largely RNA and ssDNA viruses. It is possible that the relative lack of non-tailed viruses may be the results from the way of sample preparation here, where dsDNA viruses being selected in the CsCl ultracentrifugation and library preparation for 454-based sequencing [63]. Besides, it can also be inferred as the consequence of limited non-tailed viruses in the database [62].

Additionally, the paucity of viral reference databases affects the ability to identify viral sequences in the virome. Albeit the virome analyzed in this study was revealed to be composed primarily of dsDNA viruses, the BLAST results were limited to the small fraction of sequences with matches in reference database. The unknowns could represent a great treasure or a valuable blueprint for the discovery of novel viruses as the development of bioinformatics and the continuous supplement of viral sequences. Finally, further efforts to sequence more deeply will improve our population level analysis with better assembly.

## 5. Conclusions

The present study of the JRE virome has given us a glimpse into the complexity of a virus population from a typical subtropical estuary environment. Overall, the JRE shared a similar taxonomic composition, and presented some genetic overlap with viromes from coastal environments. However, it is distinct from other publicly available datasets, representing a degree of novelty. The endemic viruses in the JRE virome were shaped by local environmental conditions, including the river, coastal ocean, and anthropogenic activities. This study provides a baseline for subsequent systemic investigations.
